# Identifying Levels of Competency in Aesthetic Medicine: A Questionnaire-based Study

**DOI:** 10.1093/asj/sjae096

**Published:** 2024-04-18

**Authors:** Sebastian Cotofana, Tristan Mehta, Kristina Davidovic, Arthur Swift, Rod J Rohrich, Brian S Biesman, Michael Gold, Andreas Nikolis, Steven Dayan, Michael Alfertshofer

## Abstract

**Background:**

In 2022, the US experienced a significant increase in demand for minimally invasive aesthetic procedures, underscoring its rising acceptance amid an unregulated educational environment for practitioners. The absence of standardized educational pathways and quality control in aesthetic medicine, primarily provided by nonacademic institutions, highlights a critical need for establishing educational standards to ensure practitioner competence and patient safety.

**Objectives:**

The aim of this study was to identify levels of competency for the aesthetic practitioner and necessary achievement milestones during the educational path from novice to expert injector.

**Methods:**

A total of *n* = 386 international study participants responded to an online questionnaire regarding their experience in aesthetic medicine practice. The questionnaire comprised 58 questions focusing on professional data, the perceived difficulty of injection, and risk for the occurrence of adverse events for specific facial regions in soft tissue filler and toxin injections.

**Results:**

Regardless of medical specialty and experience level, averages of 3.85 (1.8) years, 786.4 (2628) filler injections and 549.9 (1543) toxin injections were estimated to progress from novice to advanced injector, while averages of 6.10 (3.7) years, 1842.2 (4793) filler injections, and 1308.5 (3363) toxin injections were estimated to advance from advanced to expert injector. The nose and the perioral region have been ranked as the facial regions where it is most difficult to achieve a perfect aesthetic outcome and with the greatest risk for the occurrence of adverse events for filler and toxin injections, respectively.

**Conclusions:**

In this study we establish an educational framework in aesthetic medicine by defining the progression from novice to competent and expert injector levels, suggesting 4 years of practice and over 790 filler and 550 neuromodulator injections for competence, and at least 6 years with 1840 filler and 1310 neuromodulator injections for expertise. We also identify critical facial regions for targeted treatments by different expertise levels.

**Level of Evidence: 4:**

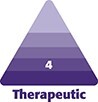

According to the annual statistics released by The Aesthetic Society in 2023, a total of 4,594,458 soft tissue filler and neuromodulator procedures were performed in 2022 in the US, representing year over year increases of 13% and 24% respectively.^1^ These increases highlight the constantly growing acceptance and procedural performance of minimally invasive treatments that are performed by physician and nonphysician aesthetic practitioners.^2,3^ Despite the growing demand for minimally invasive aesthetic procedures, the educational path for aesthetic practitioners is not standardized, monitored, or controlled by national or international boards or authorities. This is additionally affected by the fact that aesthetic medicine is predominantly practiced in the private health care sector and not within academic institutions.^4,5^

The lack of educational standardization has left aesthetic medical education in the hands of a wide array of private institutions, individual educators, and pharmaceutical companies, with the unsurprising consequence of an overwhelming number of educational options yet a complete lack of quality control. To date, no national or international accepted standards are available for aesthetic medicine education or for achievement milestones needed to help novice injectors progress to competent and subsequently to expert level. This status quo leaves an unregulated aesthetic arena in which the major source of education from the private sector may be of limited benefit for the safety of aesthetic patients. From the patient’s perspective, it would be reassuring to know that their treatment provider has achieved a certain level of competence in performance of the requested aesthetic procedure. For instance, a novice injector within their first year of aesthetic medicine practice might not be best suited to perform the more complicated and risk-bearing nasal enhancement procedures with soft tissue fillers.

The intent of the present study was to address this unmet need and provide the first step in the long process of structuring aesthetic medicine education. A questionnaire was designed to collect opinions from the aesthetic community about achievement milestones necessary for an injector in training to progress from novice to competent and from competent to expert. Additionally, the aesthetic community was queried about which facial regions portend the greatest difficulty for obtaining an ideal aesthetic outcome and the greatest risk for adverse events with soft tissue fillers and neuromodulator injections. We hoped that the survey results would help identify facial regions that are more amenable to treatments conducted by novice injectors and which facial regions should be treated predominantly by expert injectors. In the present study we define levels of competence for the aesthetic practitioner as well as demonstrable achievement milestones necessary during the educational path from novice to expert injector.

## METHODS

### Study Design

In this anonymous questionnaire-based study we collected and analyzed the answers of *n* = 386 international study participants with respect to their experience in aesthetic medicine practice. Study participants were invited to complete a previously designed online survey via social media announcements. Assuming that all social media followers of the first author (S.C.; 130k) had seen the survey announcement, the response rate to the survey was 0.3%. Only participants willing to complete the survey and provide their professional data for the analytic purposes of this study were included. Before releasing the online survey, the study received approval by the ethics committee of the University of Belgrade, Serbia, under approval no. 1A18K98AP003/2023. Data collection was conducted between January 2023 and September 2023.

### Online Survey

The online survey comprised a total of 58 questions, of which the first 4 were related to professional data and the remaining 54 were related to the core competency of the participating aesthetic practitioners. The survey was created with Google Forms (Alphabet Inc., Mountain View, CA) (Appendix, located online at www.aestheticsurgeryjournal.com).

Of the 54 questions related to aesthetic practitioner core competencies, 6 pertained to the number of years in training, the number of soft tissue filler injections, and the number of neuromodulator injections that were deemed necessary to progress from a novice to advanced and from an advanced to an expert injector. To limit investigator bias by supplying predetermined or preset answers, responses requested were free-text answers.

The remaining 48 questions were directed toward the respondents’ opinions of (1) the level of difficulty of obtaining an ideal aesthetic outcome; and (2) the perceived risk of causing an adverse event during injection with soft tissue fillers (including biostimulators) or neuromodulators for specific facial regions. Adverse events for soft tissue fillers were exemplified in the question stem as skin necrosis, soft tissue loss, or visual compromise. Adverse events for toxin injections were exemplified in the question stem as eyelid ptosis, dysphagia, or asymmetric smile.

The 15 facial regions addressed in the questionnaire for soft tissue filler injections were: forehead, glabella (= vertical glabellar lines), temples, periorbital (= A-frame deformity), tear trough, nose, medial middle face (= cheeks), lateral middle face (= zygomatic arch), nasolabial folds, mandibular angle, jawline, perioral (= smoker's lines), lips, chin, and neck.

The 9 regions addressed in the questionnaire for toxin injections were: forehead, glabella, periorbital (= lateral canthal lines), nose (= bunny lines), masseter muscle, jawline (= Nefertiti treatment, toxin lift), perioral (= treatment of levator labii superioris alaeque nasi for gummy smile, depressor anguli oris, depressor labii inferioris, orbicularis oris muscles), chin, and neck (= platysmal bands).

The answers to these 48 questions were single-choice answers based on a 5-point Likert scale. For aesthetic difficulty, the answer options included “very difficult,” “difficult,” “neutral,” “easy,” and “very easy.” For the risk of adverse events the answer options included “very low risk,” “low risk,” “neutral,” “high risk,” and “very high risk.”

### Statistical Analysis

The answers from the online questionnaire were automatically collected to a Microsoft Excel (Microsoft, Redmond, WA) spreadsheet that was downloaded and utilized for further analysis. Depending on the data structure, parametric (analysis of variance [ANOVA], 2-sided independent *t* test) and nonparametric (Spearman correlation) testing were applied. All calculations were performed with SPSS Statistics 27 (IBM, Armonk, NY), with statistical significance defined at a probability level of ≤.05 to guide conclusions.

## RESULTS

### Survey Participants

**Figure 1.**
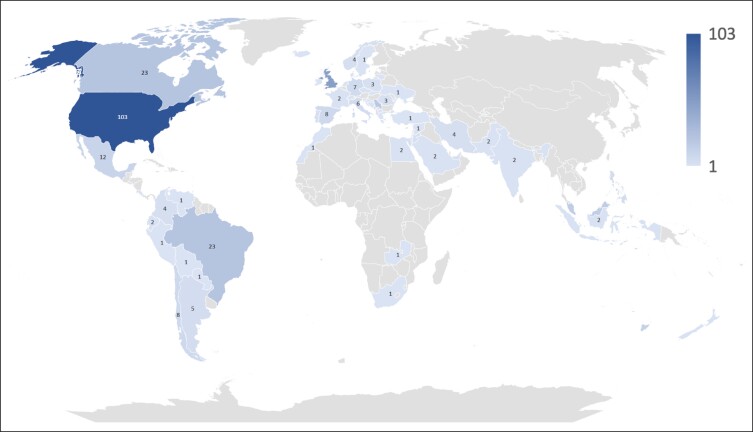
World map visualizing the country of origin of the *n* = 386 survey respondents.

**Figure 2.**
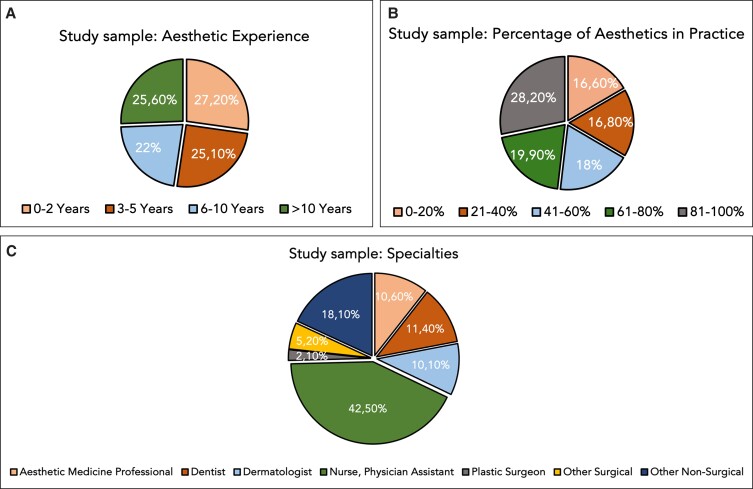
Pie charts summarizing the professional information of the study participants. (A) Experience in aesthetic medicine in years. (B) Percentage of time dedicated to minimally invasive procedures in aesthetic medicine practice during 1 week. (C) Medical specialty.

The survey was completed by *n* = 386 respondents from 61 countries (Figure 1). Of those, 2.1% were plastic surgeons, 5.2% were from other surgical specialties, 10.1% were dermatologists, 10.6% were aesthetic medicine professionals (cosmetologist, naturalist, beautician, stylist, etc), 11.4% were dentists, 18.1% were other nonsurgical specialties, and 42.5% were extender specialties (nurses, physician assistants, etc) (Figure 2).

When asked for their experience in aesthetic medicine, 27.2% stated 0 to 2 years, 25.1% stated 3 to 5 years, 22.0% stated 6 to 10 years, and 25.6% claimed more than 10 years. A statistically significant trend was observed (*P* < .001) in which plastic surgeons had the longest experience, whereas extender specialties (nurses, physician assistants) had the shortest experience (Figure 2).

When asked for the percentage of their time dedicated weekly to minimally invasive aesthetic medicine procedures, 16.6% stated 0% to 20%, 16.8% stated 21% to 40%, 18.4% stated 41% to 60%, 19.9% stated 61% to 80%, and 28.2% indicated 81% to 100%. No statistically significant difference (*P* = .106) was identified between medical specialty and the weekly percentage of time spent performing minimally invasive aesthetic procedures (Figure 2).

### Progression in Aesthetic Medicine: Years in Practice

**Figure 3.**
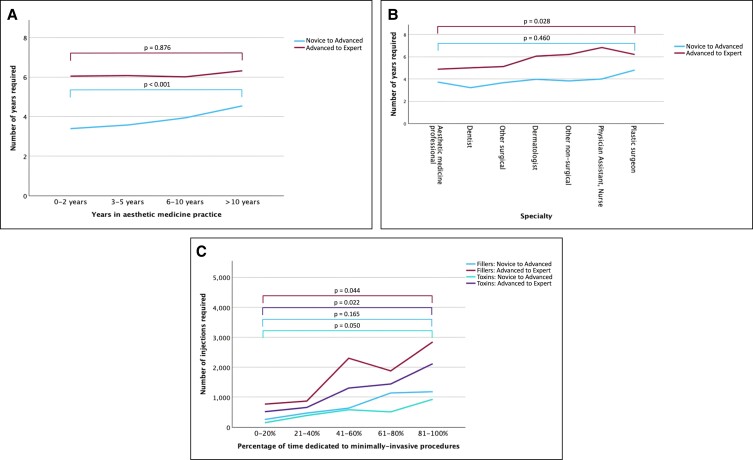
Line graphs summarizing the responses of the study participants regarding (A) years required in aesthetic medicine practice to advance from novice to advanced and from advanced to expert injector for both soft tissue filler and neuromodulator injections, by years; (B) years required in aesthetic medicine practice to advance from novice to advanced and from advanced to expert injector for both soft tissue filler and neuromodulator injections, by specialty; and (C) number of injections required to advance from novice to advanced and from advanced to expert injector for both soft tissue filler and neuromodulator injections, by percentage of time dedicated to minimally invasive procedures. Probability values were computed with 1-way analysis of variance (ANOVA) testing to compute difference across investigated categories.

When the survey participants were asked to opine on the time required to progress from a novice to advanced injector, the open answer provided by the *n* = 386 respondents was on average 3.85 (1.8) years (range: 1-10) without a statistically significant difference between specialties (*P* = .460) or between the percentages of time spent dedicated to minimally invasive procedures in aesthetic medicine practice (*P* = .079) (Figure 3).

Interestingly, when stratifying the responses for years worked in aesthetic medicine, a highly statistically significant positive correlation was identified (r_s_ = 0.271; *P* < .001), revealing that more senior aesthetic practitioners favored a longer period to transition from novice to advanced. Specifically, respondents with 0 to 2 years’ experience favored a transition at 3.42 (1.9) years, those with 3 to 5 years favored a transition at 3.57 (1.7) years, injectors with 6 to 10 years of experience favored a transition at 3.95 (1.6) years, and seasoned aesthetic practitioners with more than 10 years of experience favored a transition at 4.51 (1.9) years (Figure 3).

When asking the survey participants to determine the time needed to progress from a advanced injector to an expert injector, the answer provided was on average 6.10 (3.7) years (range: 1-25) without statistically significant difference between the respondents’ years worked in aesthetic medicine (*P* = .876) or percentages of time dedicated to minimally invasive procedures (*P* = .686). Plastic surgeons indicated that this transition should occur at 6.83 (3.5) years, whereas aesthetic medicine professionals preferred a transition to expert at 4.88 (3.2) years (*P* = .028 for differences across all specialties) (Figure 3).

### Progression in Aesthetic Medicine: Quantification of Soft Tissue Filler Injections (Including Biostimulators) Performed

When asking the survey participants to determine the number of soft tissue filler injections including biostimulators performed to progress from a novice to advanced injector, the open answers revealed an average of 786.4 (2628) (range: 1-40,000) without a statistically significant difference between specialties (*P* = .540); or the percentages of time spent dedicated to minimally invasive procedures in aesthetic medicine practice (*P* = .165); or years of experience (*P* = .464) (Figure 3).

When asking the survey participants to determine the number of soft tissue filler injections including biostimulators needed to progress from advanced to expert injector, the open answer provided was on average 1842.2 (4793) injections (range: 1-50,000) without statistically significant difference between specialties (*P* = .560) or between the varying years of experience (*P* = .864). Interestingly, respondents who spent a higher percentage of their time performing minimally invasive procedures were in favor of greater numbers of performed injections needed for progression: those who dedicated 0% to 20% of their practice to minimally invasive procedures indicated that an average of 761 injections were necessary, whereas those for whom 81% to 100% of their practice comprised minimally invasive aesthetic-based procedures indicated 2745 injections as the required number (r_s_ = 0.205; *P* < .001) (Figure 3).

### Progression in Aesthetic Medicine: Quantification of Neuromodulator Injections Performed

When asking the survey participants to determine the number of toxin injections needed to progress from a novice to advanced injector, the open answer provided was on average 549.9 (1543) injections (range: 1-20,000) with no statistically significant difference between specialties (*P* = .153), the percentages of time dedicated to minimally invasive procedures (*P* = .050), or years of experience in aesthetic practice(*P* = .518) (Figure 3).

When asking the survey participants to determine the number of toxin injections needed to progress from advanced to expert injector, the open answer provided was on average 1308.5 (3363) injections (range: 1-40,000) without a statistically significant difference between specialties (*P* = .069) or years of experience (*P* = .777). Of note, respondents who spent a greater percentage of their time performing minimally invasive procedures believed that larger numbers of injections should be performed to allow for progression: the 0% to 20% group indicated 522 injections, whereas the 81% to 100% cohort indicated 2101 injections (r_s_ = 0.174; *P* < .001) (Figure 3).

### Difficulty to Obtain a Perfect Aesthetic Outcome: Soft Tissue Fillers and Biostimulators

**Figure 4.**
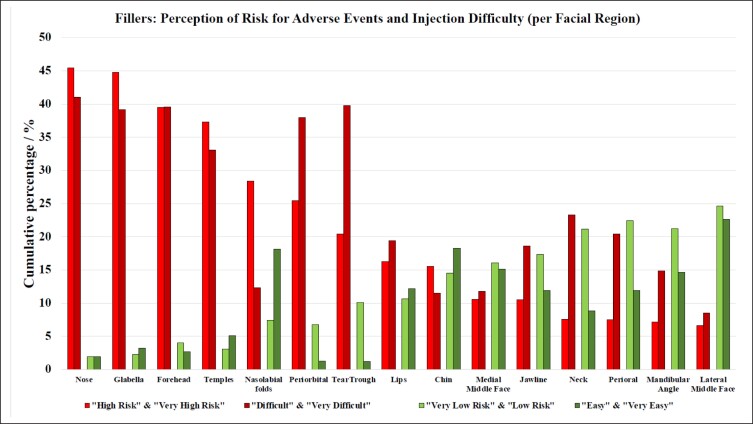
Bar graphs summarizing the perceived level of difficulty and degree of risk of adverse events in soft tissue filler (and biostimulator) injections for different facial regions. Bars are calculated as the average of both “high risk” and “very high risk”; “difficult” and “very difficult”; “very low risk” and “low risk”; and “easy” and “very easy.” The ranking is based on the percentage of answers provided for the combined score of “difficult” and “very difficult,” highest to lowest (ie, cumulative percentage).

When asking the survey participants to grade each provided facial region for the level of difficulty to achieving the perfect aesthetic outcome with soft tissue fillers or biostimulators, the following ranking was provided (ranking based on percentage of answers provided for the combined score of “difficult” and “very difficult,” highest to lowest): nose, tear trough, forehead, glabella, periorbital, temple, neck, perioral, lips, jawline, mandibular angle, nasolabial folds, medial middle face, chin, lateral middle face (Table 1, Figure 4). Interestingly, the ranking for facial regions with highest difficulty inversely corresponded at a high statistical significance with the ranking for the facial regions for highest ease (r_p_ = −0.976; *P* < .001).

**Table 1.** Ranking of Perceived Level of Difficulty of Soft Tissue Filler (and Biostimulator) Injections for Different Facial Regions

Facial region	Level of difficulty				
	Very easy	Easy	Neutral	Difficult	Very difficult
Expert injectors					
Nose	0.5%	3.4%	14.0%	32.9%	49.2%
Tear trough	0.3%	2.1%	18.1%	48.7%	30.8%
Forehead	0.0%	5.4%	15.5%	35.8%	43.3%
Glabella	0.0%	6.5%	15.3%	31.1%	47.2%
Periorbital	0.0%	2.6%	21.5%	43.0%	32.9%
Competent injectors					
Temples	1.3%	8.8%	23.8%	47.4%	18.7%
Neck	1.6%	16.1%	35.8%	34.7%	11.9%
Perioral	3.9%	19.9%	35.2%	32.1%	8.8%
Lips	4.9%	19.4%	36.8%	31.6%	7.3%
Jawline	2.1%	21.8%	38.9%	33.7%	3.6%
Novice injectors					
Mandibular angle	3.4%	25.9%	40.9%	27.2%	2.6%
Nasolabial folds	4.4%	31.9%	39.1%	21.2%	3.4%
Medial middle face	3.6%	26.7%	46.1%	21.8%	1.8%
Chin	7.3%	29.3%	40.4%	21.5%	1.6%
Lateral middle face	6.2%	39.1%	37.6%	15.5%	1.6%

The ranking was performed by averaging “difficult” and “very difficult” values for a combined score (values not shown). According to this ranking, facial regions were defined as best suited for novice, competent, and expert injectors.

### Difficulty to Obtain a Perfect Aesthetic Outcome: Neuromodulators

**Figure 5.**
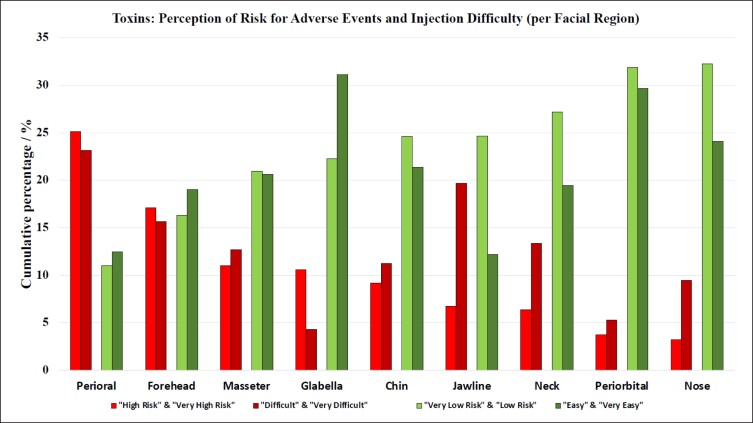
Bar graphs summarizing the perceived level of difficulty and the degree of risk of adverse events in neuromodulator injections for different facial regions. Bars are calculated as the average of both “high risk” and “very high risk”; “difficult” and “very difficult”; “very low risk” and “low risk”; and “easy” and “very easy.” The ranking is based on the percentage of answers provided for the combined score of “difficult” and “very difficult,” highest to lowest (ie, cumulative percentage).

When asked to grade each provided facial region for the level of difficulty in achieving the perfect aesthetic outcome with neuromodulators, the survey participants provided the following ranking (based on percentage of answers given for the combined score of “difficult” and “very difficult,” highest to lowest): perioral, jawline, forehead, neck, masseter muscle, chin, nose, periorbital, glabella (Table 2, Figure 5). Interestingly, the ranking for facial regions with highest difficulty inversely corresponded at a high statistical significance with the ranking for the facial regions for highest ease (r_p_ = −0.980; *P* < .001).

**Table 2.** Ranking of Perceived Risk for Causing Adverse Events With Soft Tissue Filler (and Biostimulator) Injections for Different Facial Regions

Facial region	Level of risk for adverse events				
	Very low risk	Low risk	Neutral	High risk	Very high risk
Expert injectors					
Nose	1.8%	2.1%	5.2%	43.0%	47.9%
Glabella	2.3%	2.3%	5.7%	38.3%	51.3%
Forehead	2.3%	5.7%	13.0%	46.4%	32.6%
Temples	1.3%	4.9%	19.2%	57.0%	17.6%
Nasolabial folds	1.3%	13.5%	28.5%	44.6%	12.2%
Competent injectors					
Periorbital	1.8%	11.7%	35.8%	38.6%	12.2%
Tear trough	3.1%	17.1%	38.9%	32.9%	8.0%
Lips	3.4%	17.9%	46.1%	29.0%	3.6%
Chin	4.4%	24.6%	39.9%	27.5%	3.6%
Medial middle face	4.1%	28.0%	46.6%	18.9%	2.3%
Novice injectors					
Jawline	5.7%	29.0%	44.3%	19.2%	1.8%
Neck	9.1%	33.2%	42.7%	13.0%	2.1%
Perioral	11.1%	33.7%	40.2%	13.7%	1.3%
Mandibular angle	9.1%	33.4%	43.3%	13.0%	1.3%
Lateral middle face	10.4%	38.9%	37.6%	11.4%	1.8%

The ranking was performed by averaging “high risk” and “very high risk” values for a combined score (values not shown). According to this ranking, facial regions were defined as best suited for novice, competent, and expert injectors.

### Risk for Causing Adverse Events per Facial Region With Soft Tissue Fillers and Biostimulators

When asked to grade each facial region for the perceived risk of causing adverse events, such as skin necrosis, soft tissue loss, or visual compromise, the survey participants responded with the following ranking (based on percentage of answers provided for the combined score of “high risk” and “very high risk,” highest to lowest): nose, glabella, forehead, temples, nasolabial folds, periorbital, tear trough, lips, chin, medial middle face, jawline, neck, perioral, mandibular angle, lateral middle face (Table 3, Figure 4). Interestingly, the ranking for facial regions with the highest risk inversely corresponded at a high statistical significance with the ranking for the facial regions for the lowest risk (r_p_ = −0.947; *P* < .001).

**Table 3.** Ranking of Perceived Level of Difficulty for Neuromodulator Injections for Different Facial Regions

Facial region	Level of difficulty				
	Very easy	Easy	Neutral	Difficult	Very difficult
Expert injectors					
Perioral	1.6%	23.3%	28.8%	37.8%	8.5%
Jawline	3.6%	20.7%	36.3%	35.0%	4.4%
Forehead	6.7%	31.3%	30.6%	24.6%	6.7%
Competent injectors					
Neck	6.0%	32.9%	34.5%	24.1%	2.6%
Masseter	7.8%	33.4%	33.4%	23.6%	1.8%
Chin	6.7%	36.0%	34.7%	19.9%	2.6%
Novice injectors					
Nose	9.6%	38.6%	32.9%	16.3%	2.6%
Periorbital	10.1%	49.2%	30.1%	10.1%	0.5%
Glabella	12.7%	49.5%	29.3%	7.3%	1.3%

The ranking was performed by averaging “difficult” and “very difficult” values for a combined score (values not shown). According to this ranking, facial regions were defined as best suited for novice, competent, and expert injectors.

### Risk for Causing Adverse Events per Facial Region With Neuromodulators

When grading each provided facial region for the perceived risk of causing adverse events, such as eyelid ptosis, dysphagia, asymmetric smile, or other loss of function, the survey respondents offered the following ranking (based on percentage of answers provided for the combined score of “high risk” and “very high risk,” highest to lowest): perioral, forehead, masseter muscle, glabella, chin, jawline, neck, periorbital, and nose (Table 4, Figure 5). Interestingly, the ranking for facial regions with highest risk inversely correlated at a high statistical significance with the ranking for the facial regions with the lowest risk (r_p_ = −0.972; *P* < .001).

**Table 4.** Ranking of Perceived Risk for Adverse Events of Toxin Injections for Different Facial Regions

Facial region	Level of risk for adverse events				
	Very low risk	Low risk	Neutral	High risk	Very high risk
Expert injectors					
Perioral	4.4%	17.6%	27.7%	44.0%	6.2%
Forehead	9.8%	22.8%	33.2%	31.1%	3.1%
Masseter	11.1%	30.8%	36.0%	21.0%	1.0%
Competent injectors					
Glabella	15.0%	29.5%	34.2%	19.4%	1.8%
Chin	12.2%	37.0%	32.4%	17.6%	0.8%
Jawline	13.0%	36.3%	37.3%	12.2%	1.3%
Novice injectors					
Neck	17.9%	36.5%	32.9%	11.1%	1.6%
Periorbital	19.7%	44.0%	28.8%	6.5%	1.0%
Nose	19.9%	44.6%	29.0%	5.4%	1.0%

The ranking was performed by averaging “high risk” and “very high risk” values for a combined score (values not shown). According to this ranking, facial regions were defined as best suited for novice, competent, and expert injectors.

## DISCUSSION

In this survey-based study we collected the opinions of 386 aesthetic medicine practitioners from 61 different countries with a representative participation of various medical specialties, years of experience, and relative percentage of total practice time dedicated to minimally invasive treatments. The survey was specifically designed to capture respondents’ bias-free opinions on the topic of progression of relative skill level in performing aesthetic injections by requesting free text answers instead of selecting preformed answers from a drop-down menu, because the latter would limit the study participants to selecting from a set of choices influenced by the study designers’ bias. This protocol design allowed unrestricted capture of any notions the respondents might have on the specific topic.^6^ This freedom in answering the progression (from novice to advanced and advanced to expert) questions was reflected in the data range. Looking at the maximum values for each category, some respondents suggested a temporal requirement of 10 years in aesthetic practice to progress from novice to advanced and 25 years to progress to expert. When expressed in terms of injectable experience, respondents indicated as many as 40,000 soft tissue filler injections or 20,000 neuromodulator injections be required to move from novice to advanced and that attaining expert level required 50,000 soft tissue filler injections and 40,000 neuromodulator treatments. Despite not being clinically feasible, the numbers provided represent 1 spectrum of the data range (maximum range) and not the average value; these bias-free answer options should be regarded as a strength of the questionnaire design.

A clear trend was observed when performing correlation analyses between levels of progression and responder experience, revealing that those respondents who followed a longer educational path themselves believed more experience was required to progress in aesthetic medicine (all *P* < .001): plastic surgeons, for example, indicated that reaching the level of expert injector should occur on average at 6.83 (3.5) years, whereas aesthetic medicine professionals (cosmetologist, naturalist, beautician, stylist, etc) suggested a transition to expert could occur on average after 4.88 (3.2) years. A similar trend was observed for the number of required soft tissue filler and neuromodulator injections required for progression; respondents who spent a higher percentage of their weekly time performing minimally invasive procedures favored more training. Practitioners who dedicated 0% to 20% of weekly time on these procedures suggested only 761 soft tissue filler and 522 neuromodulator injections to progress from advanced to expert injector, whereas those who dedicated 81% to 100% of their weekly time to aesthetic injections suggested an average of 2745 soft tissue filler and 2101 neuromodulator injections be performed before an injector progressed from advanced to expert level. Upon evaluation these results appeared intuitively obvious; one might anticipate that those with longer training believed others should follow a similar path. On closer consideration, however, those with greater depth of understanding can appreciate the nuances, risks, and challenges of performing injectables at a high level and their opinion may reflect this deeper understanding of vascular and muscular anatomy and ultimately patient outcomes in injectable practice.^7-14^ In clinical practice, longer training periods as well as higher numbers of procedures performed allow the practitioner to experience the wide spectrum of easy vs complex patients. This allows training on easy as well as on complex cases, improving procedural proficiency and in the long term patient safety and treatment outcome.

The survey asked aesthetic practitioners to rate the level of difficulty to achieve the perfect aesthetic outcome (if such outcome is achievable) for each facial region amenable to treatment with soft tissue fillers. The results indicated that the nose was considered most difficult, followed by the tear trough, forehead, vertical glabellar lines, and the supraorbital A-frame deformity (Table 1). Respondents were also asked to identify the facial region carrying the highest risk for potential severe adverse events associated with soft tissue filler injections and responded with the following ranking (Table 2): nose, glabella, forehead, temples, and nasolabial folds. These regions corresponded to the previously published facial high-risk zones for causing injection-related visual compromise based on the underlying vascular anatomy.^15-17^ The nose, glabella, and forehead receive direct arterial vascular supply from branches of the internal carotid artery circulation and therefore carry a substantial risk of allowing intraarterial filler product to reach the periorbital structures, including the ophthalmic artery circulation and the retina.^18-20^ The temple is a high-risk area because of communication between the zygomaticotemporal artery and the lacrimal artery, a branch of the ophthalmic artery. The nasolabial fold was likewise listed as 1 of the top 5 high-risk facial regions due to the unpredictability of the location of the facial angular artery within that region.^21,22^ These results reveal that aesthetic practitioners are aware of the high-risk regions of the face. Furthermore, they successfully indicated that the jawline, superficial neck, perioral, mandibular angle, and zygomatic arch are at lesser risk when targeted with soft tissue fillers (Figure 4).^23,24^ This again underlines the generalizability of the results obtained in this study. For neuromodulator injections, respondents classified perioral, jawline, and forehead as the most difficult facial regions (Table 3), whereas perioral, forehead, and masseter muscle were identified as high-risk regions to cause adverse events with attendant loss of normal facial function or expression (Table 4, Figure 5).

**Figure 6.**
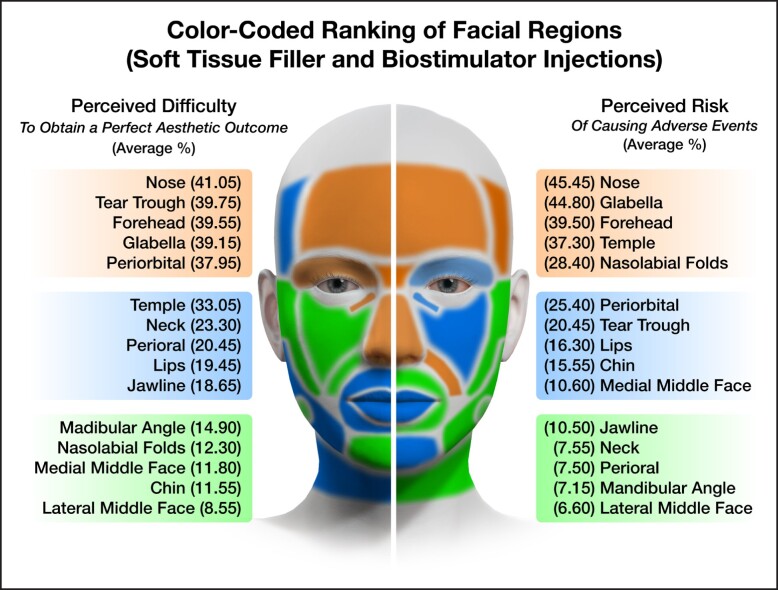
Color-coded facial map showing the various facial regions investigated in this study stratified by perceived difficulty level (right side of face) and perceived risk for causing adverse events (left side of face) when performing soft tissue filler and biostimulator injections. Green indicates facial regions best suited for novice injectors, blue facial regions best suited for competent injectors, and orange facial regions best suited for expert injectors. The percentages provided for each facial region are the cumulative percentage values of the “difficult” and “very difficult” responses as shown in Tables 1, 2.

**Figure 7.**
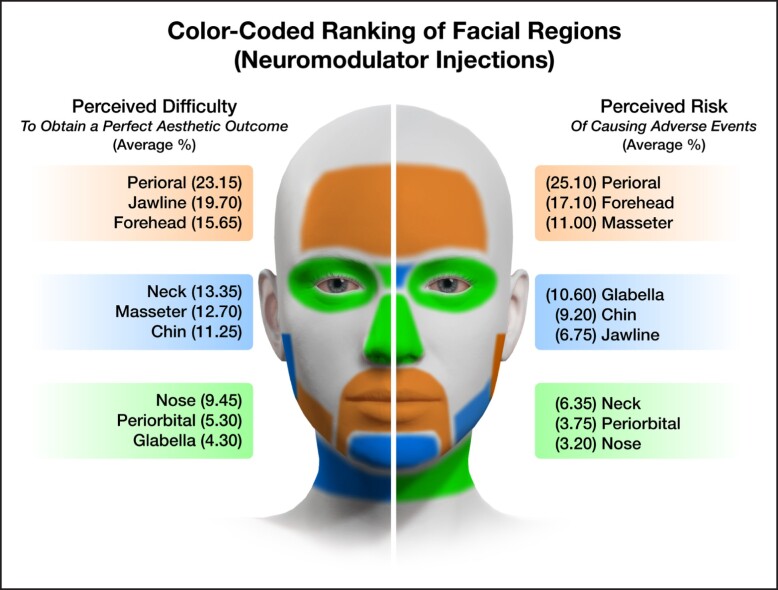
Color-coded facial map showing the various facial regions investigated in this study stratified by perceived difficulty level (right side of face) and perceived risk for causing adverse events (left side of face) when performing neuromodulator injections. Green indicates facial regions best suited for novice injectors, blue facial regions best suited for competent injectors, and orange facial regions best suited for expert injectors. The percentages provided for each facial region are the cumulative percentage values of the “difficult” and “very difficult” responses as shown in Tables 3, 4.

The results presented describe the primary analysis of the 386 international respondents. These results may suggest a starting point to identifying levels of competence in aesthetic medicine following levels of skill acquisition in the medical field, as outlined in 1980 and 1987 by Dreyfuss et al.^25,26^ The levels of skill acquisition describe the stages in which learners acquire skills through formal instruction and practice until reaching mastery in their field. The initially described 5 levels are: novice, advanced beginner, competent, proficient, and expert.^25^ However, given the complexity of 5 levels, it is suggested that, in alignment with the survey conducted, the levels of competency in aesthetic medicine be limited to 3: novice, competent, and expert, removing the intermediary levels of advanced beginner and proficient. The results presented in Tables 1 to 4 describing the difficulty and the risk for various facial regions can then be divided into thirds to assign levels of competency to the identified facial regions (see also Figures 6, 7). It is important to note that the risk level for causing adverse events and not the difficulty level for achieving an optimal aesthetic outcome determines this classification (Tables 2, 4). For instance, facial regions to be treated with soft tissue fillers by novice injectors could be the jawline, superficial neck, perioral, mandibular angle, and zygomatic arch, whereas the nose, glabella, forehead, temples, and nasolabial folds should be regions treated by expert injectors only. This regional assignment to soft tissue filler treatments is plausible and is in alignment with previous scientific evidence for facial regions most frequently associated with injection-related visual compromise.^16,17^ A similar assignment could be performed for neuromodulator injections, in which bunny lines, platysmal bands, and lateral canthal lines are facial regions potentially treated by novice injectors, whereas the perioral, forehead, and masseter muscle are to be treated by expert injectors only. To date there is no such classification available, and more studies will be needed to ascertain and provide scientific evidence for the suggestions made here. The present study is the first attempt to provide such guidance, and it can be a stepping stone toward the development of an educational framework and future research.

**Figure 8.**
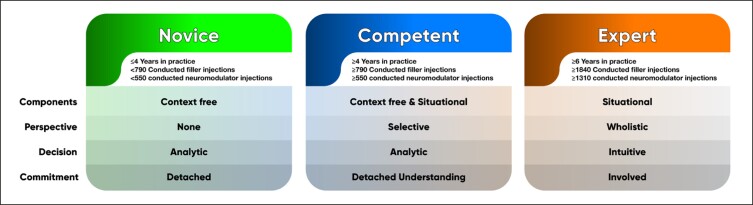
Levels of competence as identified in the study with respect to years in practice, number of performed filler injections, and number of performed neuromodulator injections.

If recommendations are to be made supporting treatment of facial regions with soft tissue filler or neuromodulator by injectors of appropriate skill level, then these skill or competency levels need to be defined. Such a definition needs to include the following domains according to the Dreyfus models: components, perspective, decision, and commitment. These 4 domains describe knowledge, situational awareness, decision-making, and the involvement of the injector in the biopsychosocial well-being of the aesthetic patient. To date no such definition has been available for the novice, competent, or expert injector; we provide it for the first time here (Figure 8):

Novice: A novice injector demonstrates a basic knowledge of anatomy (2D), rheology (soft vs hard products), and patient assessment, and can master the basic injection techniques for neuromodulators, soft tissue fillers, and biostimulators. When instructed to inject a specific facial region they can perform the required task safely and effectively. They analyze the targeted region and conduct the treatment. The novice injector has limited understanding of the resulting consequences (adverse events), how to recognize them, how to treat them, or about the biopsychosocial impact of the performed treatment (for example, feminization vs masculinization).

Competent: A competent injector has obtained advanced knowledge of anatomy (3D), various rheologic parameters (cohesivity, G-prime, G-double prime, complex modulus, etc) and patient assessment, and is able to address multiple regions with minimally invasive and noninvasive aesthetic treatment options. They analyze the region intended to treat and can foresee effects in the adjacent aesthetic regions (for example, treating the temple will affect the periorbital region). The competent injector is aware of potential adverse events and can recognize them once clinically apparent. The competent injector is also capable of understanding the effects of the conducted aesthetic treatments on the patient’s sociocultural background and understands the respective consequences for the patient (for example, a 1:1.6 lip ratio is more attractive in Caucasian than in Black or Asian cultures).

Expert: An expert injector has obtained advanced knowledge of clinically applied and functional 3D anatomy, understands the tissue integration of various products utilized depending on their rheology, can perform individualized and patient-specific assessment addressing the patient's needs, and has mastered current treatment algorithms with a plethora of minimally invasive and noninvasive aesthetic treatment options. The expert injector has also created unique personal treatment methods and can apply them effectively. The expert injector does not focus on 1 specific treatment area but always has the adjacent regions in mind and can anticipate their connections and interactions. An expert injector can extrapolate from a specific treatment outcome that can target remote regions to address the expressed aesthetic needs of a patient (for example, jawline contouring treated with temporal injections of high G-prime soft tissue fillers). An expert injector recognizes the high-risk facial regions and deploys pretreatment measures to avoid the occurrence of adverse events by conducting preinjection aspiration and ultrasound scanning, and is confident to decline a treatment if the relative risk outweighs the aesthetic benefits. Additionally, an expert injector can apply treatments that help support and positively influence the sociocultural standing of the patient and can help the patient address difficult aesthetic desires (for example, masculinization of a female or feminization of a male).

The definitions provided above are the first attempt to structure and guide the medical aesthetic field regarding educational pathways and to define milestones on the progression of an aesthetic practitioner from novice to expert. It is hoped that with the guidance of which facial regions should be treated by a novice injector and which would be better treated by more seasoned injectors, safety and treatment outcomes can be optimized and medical care can be standardized and elevated in the aesthetic community. The authors are aware that this study offers preliminary suggestions that may be incomplete and will undoubtedly undergo multiple iterations; it is a first attempt to structure aesthetic medical education based on current perceptions in the field. The survey captured the current opinion of aesthetic practitioners across the world and across various medical specialties. The results might have been more representative if members of scientific societies (International Society of Aesthetic Plastic Surgery, American Society of Plastic Surgeons, International Society for Dermatologic and Aesthetic Surgery, American Society for Dermatologic Surgery, etc) had participated and provided their insights. The current results should be seen as part of an ongoing process to structure and reflect on the educational path in aesthetic medicine. A larger sample size might have been more optimal, as would further consideration of the definition of case severity (complex vs less complex aesthetic patients); both concerns need to be regarded as limitations of this study. Another consideration is that in our survey the term “advanced” described the level between novice and expert, whereas in the Dreyfuss classification the term for this category was “competent.” The decision to change the term in our survey was based on the fact that the Dreyfuss model does not include the term advanced as a descriptor for midlevel learners, whereas in the aesthetic community the term advanced is frequently utilized and familiar to the field. It was a suitable term for the aesthetic community, as the primary target audience of the survey, was familiar with and could easily associate with a level of competency positioned between novice and expert. Another limitation of this study was the large standard deviation and data range of the results obtained when asking respondents about the numbers of injections performed necessary to progress to a higher level of competence. The standard deviation was on average 2.83-fold larger than the mean value. This may be regarded as a measure of variability in the opinion of the respondents. However, given that these questions asked for open-ended responses, the outcome may be plausible, especially because this is the first study of its kind and a plethora of heterogenous opinions are present in the aesthetic community. This very same feature may also be regarded as a strength of the study, capturing the wide variety of opinions circulating in the field.

## CONCLUSIONS

In this study we collected and analyzed the answers of an online survey completed by 386 international respondents with various medical aesthetic backgrounds and varied levels of dedication to the practice of aesthetic injections. The results revealed that more experienced aesthetic practitioners favored a longer educational path for novice injectors to progress to the next level of competency, with a benchmark of 4 years of aesthetic practice, 790 soft tissue filler injections, and 550 neuromodulator procedures performed. To reach the highest level of injector, expert, more experienced practitioners surveyed indicated that at least 6 years of aesthetic practice, 1840 soft tissue filler procedures, and 1310 neuromodulator injections should be accomplished. The results can also be utilized to define facial regions best targeted by novice injectors and those best targeted by expert injectors (eg, the nose, glabella, forehead, temples, and nasolabial folds for soft tissue filler injections). This study additionally provided for the first time a definition of novice, competent, and expert injectors as defined by the standard levels of skill acquisition. It is hoped that the results presented here will guide implementation of a structured educational path in plastic surgery (or other medical specialty) residency programs to increase training and ultimately proficiency with injectable treatments.

## Supplemental Material

This article contains supplemental material located online at www.aestheticsurgeryjournal.com.

## Supplementary Material

sjae096_Supplementary_Data
