# Supplementary material for: Identifying Levels of Competency in Aesthetic Medicine: A Questionnaire-based Study
Source: Aesthet Surg J. 2024 Apr 18;44(10):1105–17. doi: 10.1093/asj/sjae096 (PMC11403812; doi:10.1093/asj/sjae096)
Supplement: sjae096_Supplementary_Data [file sjae096_supplementary_data.pdf]

Survey: Identifying levels of difficulty for facial aesthetic treatments – A survey-based study

Dear Study Participant,
Thank you for taking the time to complete the following questions. Your anonymized answers will be used for a scientific publication which will help to guide aesthetic medicine practitioners worldwide. No data will be collected, stored or distributed to a third party. Your anonymized responses will be deleted after the data is extracted and used for statistical analyses. If you do not wish to participate, please close the survey.
Thank you very much!

Background of this study: Starting an aesthetic career is difficult. A plethora of anatomic knowledge, injection techniques and product information needs to be learned, understood and applied. In addition, the first treatments need to be catered to the individual skill level. However, to date there are no guidelines available which facial regions can be targeted by novice injectors and which facial region is better suited to be treated by expert injectors.

This study was therefore designed to guide novice injectors towards safer treatments. Novice injectors should receive guidance which facial region carries a lower risk or a greater risk when it comes to difficulties in aesthetic outcomes and safety.

Thank you for answering the following 14 questions!

scotofana24@gmail.com Switch account

Not shared

* Indicates required question

Please indicate in which country you practice aesthetic medicine. *

Your answer

Please indicate your years of experience in aesthetic medicine. *

- 0-2 years
- 3-5 years
- 6-10 years
- >10 years

Please indicate the percentage of time you dedicate on a weekly basis towards performing minimally-invasive aesthetic procedures (= injectable treatments). *

- 0-20%
- 21-40%
- 41-60%
- 61-80%
- 81-100%

Please indicate your medical specialty. *

- Aesthetic Medicine Professional (Cosmetologist, Naturalist, Beautician, Stylist etc.)
- Nurse, Physician Assistant (Other Extender Specialties)
- Dentist
- Dermatologist
- Plastic Surgeon
- ENT/Facial Plastic Surgeon
- Ophthalmologist/Oculoplastic Surgeon
- Other non-surgical
- Other surgical

Please indicate what you believe is the minimum number of years of training required to progress from **NOVICE** to **ADVANCED** injector**.** *
Please provide a number.

Your answer

Please indicate what you believe is the minimum number of performed soft tissue filler injections (including biostimulators) required to progress from **NOVICE** to **ADVANCED** injector**.** *
Please provide a number.

Your answer

Please indicate what you believe is the minimum number of performed toxin injections required to progress from **NOVICE** to **ADVANCED** injector**.** *
Please provide a number.

Your answer

Please indicate what you believe is the minimum number of years of training required to progress from **ADVANCED** to **EXPERT** injector**.** *
Please provide a number.

Your answer

Please indicate what you believe is the minimum number of performed soft tissue filler injections (including biostimulators) required to progress from **ADVANCED** to **EXPERT** injector**.** *
Please provide a number.

Your answer

Please indicate what you believe is the minimum number of performed toxin injections required to progress from **ADVANCED** to **EXPERT** injector**.** *
Please provide a number.

Your answer

Please indicate the level of difficulty for obtaining a perfect **aesthetic outcome** for treatments with **soft tissue fillers** (including biostimulators) in each of the following facial regions. *

	Very difficult	Difficult	Neutral	Easy	Very Easy
Forehead	○	○	○	○	○
Glabella	○	○	○	○	○
Temple	○	○	○	○	○
Periorbital (A-frame deformity)	○	○	○	○	○
Tear Trough	○	○	○	○	○
Nose	○	○	○	○	○
Medial Midface (cheeks)	○	○	○	○	○
Lateral Midface (zygomatic arch)	○	○	○	○	○
Nasolabial fold	○	○	○	○	○
Mandibular angle	○	○	○	○	○
Jawline	○	○	○	○	○
Perioral (Smoker's lines)	○	○	○	○	○

Lips	◯	◯	◯	◯	◯
Chin	◯	◯	◯	◯	◯
Neck	◯	◯	◯	◯	◯

Please indicate the level of difficulty for obtaining a perfect **aesthetic outcome** for treatments with **neuromodulators** in each of the following facial regions. *

	Very difficult	Difficult	Neutral	Easy	Very Easy
Forehead	○	○	○	○	○
Glabella	○	○	○	○	○
Periorbital (Crow's feet)	○	○	○	○	○
Nose (Bunny lines)	○	○	○	○	○
Masseter muscle	○	○	○	○	○
Jawline (Toxin lift)	○	○	○	○	○
Perioral (DAO, Lip flip, Gummy smile)	○	○	○	○	○
Chin	○	○	○	○	○
Neck (Platysmal bands)	○	○	○	○	○

Please indicate your personally perceived risk for having an **adverse vascular event** (e.g., skin necrosis, soft tissue loss, visual compromise) after the treatments with **soft tissue fillers** (including biostimulators) in each of the following facial regions. *

	Very low risk	Low risk	Neutral	High risk	Very high risk
Forehead	○	○	○	○	○
Glabella	○	○	○	○	○
Temple	○	○	○	○	○
Periorbital (A-frame deformity)	○	○	○	○	○
Tear Trough	○	○	○	○	○
Nose	○	○	○	○	○
Medial Midface (cheeks)	○	○	○	○	○
Lateral Midface (zygomatic arch)	○	○	○	○	○
Nasolabial fold	○	○	○	○	○
Mandibular angle	○	○	○	○	○
Jawline	○	○	○	○	○
Perioral (Smoker's	○	○	○	○	○

lines)

Lips	○	○	○	○	○
Chin	○	○	○	○	○
Neck	○	○	○	○	○

Please indicate your personally perceived risk for having **adverse events** (e.g., eyelid ptosis, dysphagia, asymmetric smile, etc.) after the treatments with **neuromodulators** in each of the following facial regions. *

	Very low risk	Low risk	Neutral	High risk	Very high risk
Forehead	○	○	○	○	○
Glabella	○	○	○	○	○
Periorbital (Crow's feet)	○	○	○	○	○
Nose (Bunny lines)	○	○	○	○	○
Masseter muscle	○	○	○	○	○
Jawline (Toxin lift)	○	○	○	○	○
Perioral (DAO, Lip flip, Gummy smile)	○	○	○	○	○
Chin	○	○	○	○	○
Neck (Platysmal bands)	○	○	○	○	○

Thank you for participating in this survey!

Submit Clear form

Never submit passwords through Google Forms.
